# Multifaceted Roles of Connexin 43 in Stem Cell Niches

**DOI:** 10.1007/s40778-018-0110-3

**Published:** 2018-02-15

**Authors:** Nafiisha Genet, Neha Bhatt, Antonin Bourdieu, Karen K. Hirschi

**Affiliations:** 1 Department of Medicine, Genetics and Biomedical Engineering, Yale Cardiovascular Research Center, Vascular Biology Therapeutics Program, New Haven, USA; 2 Yale Stem Cell Center Yale University School of Medicine, 300 George St, New Haven, CT 06511 USA

**Keywords:** Connexin 43, Stem cell niches, Cell-to-cell interactions, Vascular endothelial cells

## Abstract

**Purpose of Review:**

Considerable progress has been made in the field of stem cell research; nonetheless, the use of stem cells for regenerative medicine therapies, for either endogenous tissue repair or cellular grafts post injury, remains a challenge. To better understand how to maintain stem cell potential in vivo and promote differentiation ex vivo, it is fundamentally important to elucidate the interactions between stem cells and their surrounding partners within their distinct niches.

**Recent Findings:**

Among the vast array of proteins depicted as mediators for cell-to-cell interactions, connexin-comprised gap junctions play pivotal roles in the regulation of stem cell fate both in vivo and in vitro.

**Summary:**

This review summarizes and illustrates the current knowledge regarding the multifaceted roles of Cx43, specifically, in various stem cell niches.

## Introduction

Cell-to-cell communication is required to coordinate and maintain physiologic cellular activities within tissues. Gap junction (GJ) channels that form between juxtaposed membranes of two adjacent cells connect their cytosols and enable the intercellular transfer of ions, metabolites, soluble factors, and messenger molecules (< 1 kDa), which regulate signaling pathways needed for cell survival, proliferation, and fate decisions. This type of intercellular communication is thought to play an important role in stem cell niches to regulate stem and progenitor cell activation and proliferation. However, although the existence of GJ in adult niches, such as in bone marrow, has been known for over 30 years, their function(s) is not clearly defined. In fact, functional studies aimed at understanding the role of connexin-comprised GJ in the bone marrow and neural as well as skin stem cell niches are all underway, and we will provide an overview of their common and unique role(s) in the regulation of stem and progenitor cell survival, proliferation, and fate in these distinct microenvironments.

## Connexins, Connexons, and Gap Junctions

**Fig. 1:**
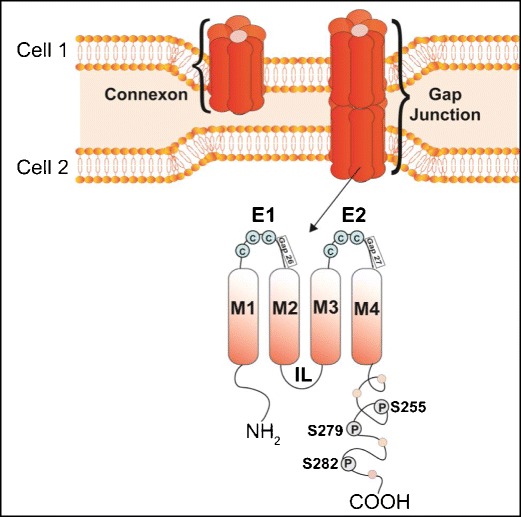
Structural organization of a connexin (Cx) protein, a connexon hemichannel, and a gap junction. One connexon or hemi-channel is formed by the association of six connexin (Cx) proteins. Two hexameric connexons between two adjoining cells dock to form a gap junction channel. Topologically, one Cx is composed of four transmembrane domains (M1 to M4), two extracellular loops (E1 and E2), one intracellular loop (IL), and intracellular amino (NH_2_) and carboxy (COOH)-termini. E1 and E2 have three cysteine residues (C) that form disulfide bonds with adjoining Cx proteins, allowing the docking of two connexons. The Cx-mimetic blocking peptides Gap26 and Gap27 specifically target E1 and E2, respectively. The C-terminal is subjected to a variety of post-translational modifications, including phosphorylation at the S255, S279, and S282 sites by the mitogen-activated protein kinases (MAPK)

Connexins (Cx) are highly conserved proteins both structurally and topologically. They consist of four transmembrane domains (M1, M2, M3, and M4), one intracellular loop (IL), and two extracellular loops (E1 and E2); both the N- and C-termini are cytoplasmic [1, 2] (Fig. 1). Cx proteins are first synthetized in the endoplasmic reticulum and the hexameric oligomerization of six Cx into a connexon takes place in the trans-Golgi network. The connexon or hemi-channel is then transported to the plasma membrane where it can dock to another hemi-channel of an adjoining cell to form an intercellular, or GJ channel. The docking of two hemi-channels requires the formation of disulfide bonds between three cysteine residues on the extracellular loops E1 and E2 of each Cx (Fig. 1). Many GJ then assemble together to form GJ plaques [3]. Newly synthetized connexons are incorporated on the outer edges of the junctional plaque, while older GJ simultaneously migrates toward the center of the plaque where they are internalized into connexosomes for degradation. Degradation processes involve the proteasome, lysosome, phago-lysosomal, and autophagy pathways [4–8]. GJ channels within plaques have a high turnover rate since Cx protein half-lives are relatively short, ranging from 1.5 to 5 h [9]. The modulation of the Cx turnover rate represents an important mechanism by which cells regulate GJ intercellular coupling (GJIC) [10].

The complex Cx gene family comprises 21 isoforms in mice and 20 isoforms in humans; 19 of them are orthologous pairs due to their sequence identity. Hemi-channels can be formed by identical Cx isoforms, known as homomeric connexons, or by combination of different Cx isoforms assembling into heteromeric connexons. The association of two homomeric connexons forms a homotypic GJ, and the docking of either one homomeric with one heteromeric connexon or two heteromeric connexons forms heterotypic GJ channels. [11]. In this review, we will focus only on Cx43, as it is the most highly expressed in stem cell niches and the best characterized Cx protein.

## Channel-Dependent and Channel-Independent Functions of Cx

### Channel-Dependent Functions

The formation of intercellular GJ channels is highly dependent on the bonding of cysteine residues in the extracellular loops of Cx proteins in juxtaposed hemi-channels. The hydrophilic pore of GJ channels is formed by the third transmembrane domain (M3) due to its high content in negatively and positively charged residues [12]. GJIC is highly regulated by the number of GJ channels present within the cellular membranes, their functional state (open vs. closed), and selectivity of molecules that traverse the channels. The functionality of GJ channels is dependent on the phosphorylation state of the Cx proteins that comprise the channels, and various other factors such as Ca^2+^, O_2_, shear stretch, pH, voltage, and protein to protein interactions [13]. Several tools have been developed to study GJIC, or channel-dependent function of Cx. They include drugs that close GJ channels, such as heptanol, octanol, 18α-glycyrrhetinic acid, carbenoxolone, and oleamide; however, these reagents are non-specific pharmacological inhibitors [14–16]. More GJ-selective regulation can be mediated via synthetic Cx-mimetic blocking peptides, such as Gap26 and Gap27, which are homologous to specific amino acid sequences in the EC loops E1 and E2, respectively [17–20] (Fig. 1). Genetic approaches using single site mutations of specific amino acids in the Cx sequence are alternate and robust tools to study GJ channel function. In addition, the threonine (T) residue located at the cytoplasmic end of M3 is conserved among the Cx proteins; mutating the T residue into an alanine (A) results in a “closed channel” state. For example, generating the Cx43T154A mutation in Cx43 renders its channels non-functional, despite the fact that channels retain their capacity to localize properly within the plasma membrane and form GJ plaques [21].

As mentioned earlier, the Cx gene family is highly conserved; however, the length and sequence of the cytosolic C-terminus or carboxy tail (CT) are different from one Cx to another. The CT provides sites for protein to protein interactions and is subjected to extensive post-translational modifications that regulate Cx intracellular trafficking and GJ channel gating [22]. Post-translational modifications include acetylation, *S*-nitrosylation, ubiquitination, SUMOylation, and phosphorylation. Phosphorylation of the Cx43 CT tail controls GJIC through GJ channel gating, Cx trafficking, assembly of connexons into GJ, GJ endocytosis, and degradation [22]. Cx phosphorylation is often triggered in response to the activation of receptor protein tyrosine kinases. Cx43 is mainly phosphorylated on serine (S) residues but can also be phosphorylated on tyrosine and threonine residues. Some sites when phosphorylated enhance GJIC (activating phospho-sites), while others can inhibit GJIC (inhibitory phospho-sites). For example, epidermal growth factor (EGF) inhibits GJIC by activating MAPK-dependent phosphorylation of Cx43 at S255, 279, and 282 [23–26]. Platelet-derived growth factor (PDGF) inhibits GJIC via MAPK and PKC signaling cascades. Additionally, PDGF and EGF can induce the endocytosis and degradation of Cx43 [27–30]. Proteins enhancing GJIC include casein kinase (CK1) and cAMP. CK1 promotes GJ assembly by initial phosphorylation of Cx43 at S325, 328, and 330 followed by S306 or 314 in a hierarchical fashion [31]. Elevated cytoplasmic levels of cAMP enhance GJIC via protein kinase A (PKA) and increase trafficking of Cx43 to the plasma membrane and GJ assembly [32–34].

### Channel-Independent Functions

There is increasing evidence that the Cx43 CT interacts with other proteins to modulate intracellular signaling, thus allowing Cx43 to regulate cell proliferation, differentiation, and migration, independent of its ability to form functional intercellular GJ channels. Cancer researchers have highlighted the importance of the Cx43 CT in the regulation of tumor cell growth, independent of GJ formation [35–38]. For example, Huang and coworkers transfected Cx43 CT into glioma cells, which suppressed tumor growth, without establishing GJIC [39]. In addition, enforced expression of the Cx43 CT in mouse neuroblastoma cells was sufficient to induce growth arrest to the same extent as the full-length, wild-type (WT) Cx43 [36]. Finally, stable overexpression of the Cx43 CT in HeLa cells significantly decreased proliferation; in these cells, the CT fragment was localized to the cytoplasm and the nucleus, providing further evidence that plasma membrane localization and channel formation are not required for Cx-mediated growth control [40].

Regarding mechanism(s) of action, the Cx43 CT promotes the degradation of the S-phase kinase-associated protein 2 (Skp2), which, in turn, upregulates p27 and subsequently arrests cell growth [41]. Moreover, the Cx43 CT also upregulates the expression of p53, a potent tumor suppressor [42, 43••]. The Cx43 CT also functions as a signaling hub/interactome, and β-catenin is among the interacting partners involved in its GJIC-independent function. Interaction of the Cx43 CT with β-catenin suppresses downstream Wnt-mediated regulation of proliferation, migration, and apoptosis [44, 45]. The CT of Cx43 also interacts with zonula occludens (ZO)-1 and ZO-2 at their PDZ-binding motifs, as well as α and β tubulin, the main components of microtubules [46].

The use of genetically malleable mouse models has also shed light on the in vivo role of the Cx43 CT. For example, mice carrying the mutation Cx43K258stop, in which the last 125 amino acid residues of the CT have been deleted, die shortly after birth due to defective epidermal barrier, associated with impaired terminal differentiation of keratinocytes. Haplo-insufficient K258stop/Cx43KO mice, carrying one K258stop and one Cx43 KO allele, make functional GJ despite the absence of any full-length Cx43, but the truncation of the CT affects the localization of Cx43 and reduces the number of junctional plaques in vivo [47]. These mice also have altered neuronal migration in the neocortex, comparable to that observed in Cx43 KO mice [48]. The CT has also been shown to be an important mediator of neuroprotection during cerebral ischemia by enhancing astrogliosis after stroke [49]. More recently, the Cx43 CT has been shown to bind to multiple signaling proteins needed for optimal osteoblast function, namely PKCδ, ERK, and β catenin [50••]. As a result, the K258stop/Cx43KO mice have several defects in osteoblast proliferation, differentiation, and collagen deposition [51•]. This clearly emphasizes the importance of the Cx43 CT, possibly as a docking hub for multiple proteins needed for different signaling cascades regulating cell function, and this may be relevant to Cx43 regulation of stem and progenitor cells.

## Cx43 in Stem Cell Niches

### Definition of Adult Stem Cell Niches

The concept of a specialized microenvironment, or niche, for stem cells was first proposed by Schofield more than 3 decades ago [52]. At that time, he suggested that niches have a distinct anatomical location within tissues and that removal of stem cells from their niches induces differentiation. Since then, mammalian stem cell niches have been described in multiple organ systems and shown to provide structural support and molecular cues that determine stem cell quiescence, self-renewal, and activation. Therefore, the niche is not only a physical location for stem cells, but a hub where various extrinsic signals are integrated to regulate intrinsic stem cell behavior. These cues include cell-to-cell contacts, cell-to-matrix interactions, and autocrine and paracrine-soluble effectors. In this second part of the review, we will focus on the role of indirect and direct cellular interactions among niche cells in various stem cell niches and, more specifically, on interactions mediated via Cx43-hemi-channels and Cx43-containing GJ channels.

### Hematopoietic Stem Cell Niche: Bone Marrow

**Fig. 2:**
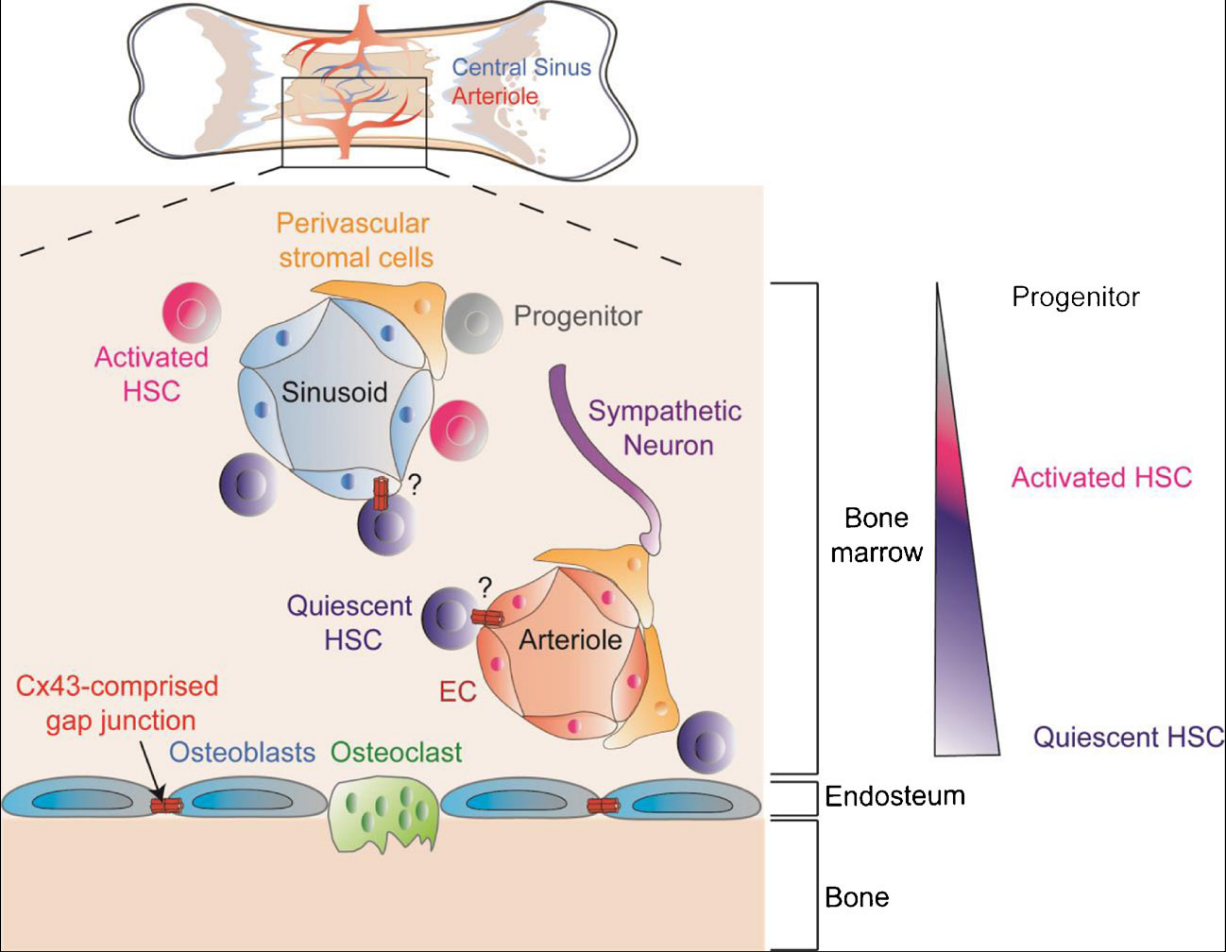
Cellular architecture of the hematopoietic stem cell (HSC) niche in the adult bone marrow. The osteoblastic niche resides along the endosteum, located at the periphery of the bone, while the vascular niche is centrally localized in the bone marrow. HSC are localized adjacent to sinusoids and arterioles. The central vascular niche is thought to favor activation and differentiation of HSC. Cx43-comprised gap junctions between osteoblasts regulate HSC behavior within the niche. Although vascular endothelial cells and HSC express Cx43, the existence, and physiologic role, of Cx43-mediated interactions between vascular endothelial cells and HSC remains to be determined

Adult hematopoietic stem cells (HSC) reside in the bone marrow, where they constantly self-renew, proliferate, and differentiate to enable the production of mature blood cell types throughout life. The critical cellular components of the bone marrow hematopoietic niche are still under debate and intense investigation. At present, the field suggests two types of niches: (1) the osteoblastic niche and (2) the vascular niche [53, 54••] (depicted in Fig. 2). It is likely that quiescent HSC reside in the osteoblastic niche, which is located along the peripheral bone endosteal region [55–57]. In the centrally localized vascular niche, thought to favor differentiation, HSC lie in close proximity to sinusoidal blood vessels, arterioles, and perivascular cells [55–65]. Similarly, the endosteal region is equally vascularized, which implicates vascular endothelial cells as potential regulators of HSC behavior in both the osteoblastic and vascular niches.

Several studies have delineated the roles of cell-to-cell interactions between HSC and surrounding endothelial cells, stromal cells, and osteoblasts in the regulation of adult HSC “stemness” capacity. In the osteoblastic niche, HSC and osteoblasts interact via Wnt-β catenin signaling and Notch signaling to maintain HSC [53, 66–75]. In addition, the existence of functional GJ has also been reported in the bone marrow niche, wherein Cx43 is the most highly expressed Cx [76–80]. Several groups have studied the role of Cx43-mediated interactions between bone marrow stromal cells and osteoblasts in the regulation of HSC. For example, Wagner and coworkers identified Cx43 as an important regulator of HSC self-renewal [81]. In bone marrow stromal cells, Cx43 also exists within hemi-channels that are not docked with adjacent cells and trigger the release of ATP, activating purinergic receptors, which upon activation increases HSC growth and potential [80]. Several in vitro studies reported that bone marrow stromal cells expressing Cx43 regulate hematopoiesis. In the S17 stromal cell line co-cultured with HSC, overexpression of Cx43 delays differentiation of blood cells, while loss of Cx43 accelerates myelopoiesis [82].

The vascular niche harbors sinusoidal blood vessels and arterioles that control HSC maintenance. It has been shown that sinusoidal endothelial cells support HSC quiescence and self-renewal through the paracrine secretion of the growth factor pleitrophin (PTN) and the angiocrine factor Jagged1 [83, 84]. Conversely, direct contact between endothelial cells and HSC via endothelial-expressed E-selectin promotes HSC proliferation [85]. In the arteriolar niche, perivascular cells expressing the chondroitin sulfate proteoglycan neural/glial antigen NG2 maintains HSC quiescence and reduces the long-term repopulating capacity of HSC [65].

Although Cx43-deficient (Cx43^−/−^) mice die shortly after birth due to heart malformations [86], precluding postnatal hematopoietic studies, embryonic Cx43^−/−^ mice exhibit reduced fetal liver HSC and progenitor cells. Cx43 deficiency in stromal cells, specifically, results in a reduction of functional HSC and progenitor cells in the fetal liver and impairs the growth and differentiation of bone marrow HSC, suggesting that Cx43 acts as critical regulator of hematopoiesis [87, 88]. Furthermore, Cx43 was shown to be critical for injury-induced hematopoietic regeneration in adults [89]. Cx43 is thought to prevent irradiation-induced mitochondrial superoxide anion (O_2_^·^) accumulation in the HSC [90]; hence, Cx43 protects HSC and progenitor cells from O_2_^·^-induced senescence, allowing them to replace lost HSC following injury [91, 92]. Whether Cx43-mediated contacts between HSC and vascular endothelial cells in the bone marrow niche are critical for HSC regulation remains unknown, opening new avenues for investigation.

### Neural Stem Cell Niches

**Fig. 3:**
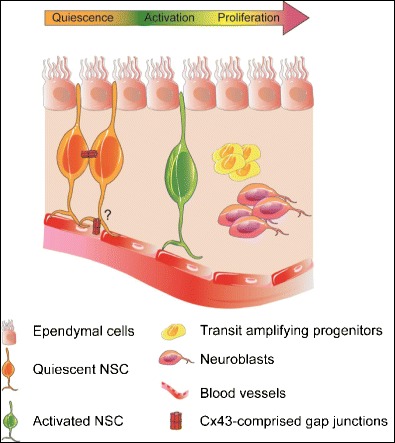
Cellular architecture of the adult brain subventricular zone (SVZ) niche. The SVZ is a polarized niche with a network of quiescent and activated neural stem cells (NSC) extending basal end-feet on endothelial cells of blood vessels, while protruding apical processes through the layer of ependymal cells that line the lateral ventricles. When neurogenesis is initiated in the SVZ, activated NSC rapidly divide into transit-amplifying progenitors that give rise to neuroblasts. It is thought that Cx43-containing gap junctions among quiescent NSC regulate NSC behavior. Whether Cx43- and gap junction-mediated interactions between NSC and endothelial cells, or ependymal cells, exist and contribute to NSC regulation remains to be determined

Neural stem cells (NSC) and progenitor cells reside in two neurogenic regions of the adult mammalian brain: the subventricular zone (SVZ) lining the walls of the lateral ventricles (depicted in Fig. 3) and the subgranular zone (SGZ) of the dentate gyrus (DG) in the hippocampus. Within these two neurogenic niches, NSC display self-renewal capacity, and the ability to differentiate into glial (astrocytes and oligodendrocytes) and neuronal (granular and periglomerular neurons) lineages. These brain regions support ongoing neurogenesis throughout development and postnatally.

### Subventricular Zone

In the SVZ, NSC reside in the subependymal region, separated from the cerebral spinal fluid (CSF)-filled ventricles by a layer of ciliated ependymal cells (also called Type-E cells). The SVZ functions as a neurogenic environment maintaining NSC in a poised and undifferentiated state, ready to react to changes in the microenvironment. Within the niche, quiescent or slow-proliferating radial-glial-like NSC (also called Type-B cells) are “anchored” by two distinct cell types [93]. On the ventricular side, they are attached to ependymal cells and protrude apical processes through the ependymal cell layer, into CSF; on the parenchymal side, they project specialized basal-end feet toward endothelial cells of blood vessels [94–97]. The apical processes arising from a Type-B cells are surrounded by rosette-like structures formed by two types of ependymal cells: multi-ciliated Type-E1 cells controlling CSF flow and Type-E2 cells composed of two cilia and complex basal bodies. Together, quiescent Type-B cells and ependymal cells form pinwheel structures [96].

The process of neurogenesis is initiated in the SVZ when Type-B cells are activated and become fast dividing progenitors, named transit-amplifying, or Type-C, cells. Type-C cells then form small clusters and differentiate into migrating neuroblasts, called Type-A cells [98]. Type-A cells continue to proliferate but ten times slower than Type-C cells [93, 99]. Type-A neuroblasts migrate from the anterior part of the SVZ along the rostral migratory stream toward the olfactory bulb and eventually differentiate into granular and periglomerular neurons [100–102].

In the SVZ, the blood-brain barrier is “leaky,” with vascular endothelial cells lacking astrocyte end-feet and pericytes coverage, thus enabling direct interactions between NSC and endothelial cells via adhesion molecules, tight junctions, and GJ, which are thought to regulate NSC self-renewal and potential [103–111]. The SVZ microvasculature is thought to be an integral component of the NSC niche and is suggested to support NSC self-renewal, maintenance, proliferation, as well as differentiation and migration of neural progenitor cells [95–97]. Cx43 is highly expressed by NSC and vascular endothelial cells in the SVZ, and although the role of GJIC between these cell types is unclear, it is known that Cx43 plays key roles in (1) maintaining neural progenitor cells in non-differentiated and proliferative states [112, 113], (2) promoting neuroblast migration [114, 115], and (3) maintaining the cellular architecture of the SVZ niche [116]. It was demonstrated that Cx43 channel activity stimulates neural progenitor cell proliferation by synchronizing Ca^2+^ activity and regulating intracellular Ca^2+^ homeostasis [117, 118]. In early post-natal SVZ, cell cycle progression of NSC requires an increase in blood flow in the niche, which is under the control of neuro-metabolic vascular coupling provided by ATP release from neural progenitor cells [119, 120]. Cx43 hemi-channel activity might contribute to the mechanisms required for adequate blood flow to regions of extensive cell proliferation through the release of vasoactive substances triggering the local hemodynamic responses [80].

### Subgranular Zone of the Dentate Gyrus

Within the DG of the hippocampus, NSC reside in the subgranular zone (SGZ) between the granule layer and the hilus. In the DG, Type-B radial glia-like cells are thought to represent the NSC population that can generate proliferating intermediate progenitor cells (Type-D cells) with transit-amplifying characteristics [102]. These Type-D cells progressively generate more differentiated progeny and migrate a short distance to the granule layer, where they differentiate into mature granule neurons (Type-G cells) and integrate the hippocampal circuits [107, 121, 122]. Type-B cells have basal processes that contact blood vessels beneath the DG, as well as apical processes that contact the granule layer, and there is a close relationship between proliferating neural progenitors and endothelial cells in the hippocampus [123]. In the SGZ, neurogenesis is coupled to angiogenesis, as suggested by high levels of vascular endothelial growth factor (VEGF) and vascular endothelial growth factor receptor 2 (VEGFR2) [124]. More recently, it has been demonstrated that VEGFR3 controls NSC activation in hippocampal neurogenesis, and VEGFR3::YFP mice demonstrates VEGFR3 expression in NSC [125•].

In addition to VEGF signaling, there is growing evidence implicating cell-to-cell interactions mediated by Cx43-containing GJ in hippocampal neurogenesis. In the post-natal hippocampus, neural progenitors are strongly coupled by GJ comprised of Cx43 [126, 127], and deletion of Cx43 in vivo affects hippocampal neurogenesis [128]. This observation was further confirmed by Liebman and coworkers, who demonstrated that Cx43 promotes the survival of newborn neurons [129]. Cx43 has also been shown to be upregulated in the DG following traumatic brain injury, enhancing post-injury neurogenic response [130••]. Single-cell RNA-seq and bioinformatic approaches demonstrated reduced cell-to-cell communications as quiescent NSC progress toward an activated cycling state; tight junction protein 2 (ZO-2) and GJA1 (Cx43) were among the downregulated genes [131••]. It is possible that during this process, Cx43 is not forming GJ channels but is required to stabilize other junctional proteins, via the CT. The expression of Cx43 in vascular endothelial cells in the DG has not been reported, and whether the vasculature influences hippocampal neurogenesis through Cx43-containing GJ remains to be investigated.

### Skin Stem Cell Niche

The skin is a complex organ harboring distinct populations of stem cells and is layered with epidermis, dermis, and subcutaneous fat, the dermis being the outermost layer. Stem cell populations within the skin include epidermal, hair follicle, and melanocyte stem cells [132]. In adult skin, hair follicles undergo repeated cycles of apoptosis (catagen) and regeneration (anagen) followed by a rest period (telogen) [132–134]. Slow-cycling hair follicle stem cells (HFSC) reside in a specialized “bulge” pocket, located at the top of the follicle that does not degenerate throughout the catagen phase of the hair cycle. HFSC are subdivided into two populations, quiescent stem cells located in the bulge (Bu-SC) and primed stem cells located beneath the bulge within the hair germ, which are more prone to proliferation. During the anagen phase, hair germ develops into a matrix of fast-cycling transit-amplifying progenitors followed by a terminal differentiation forming the hair shaft. In contrast, Bu-SC give rise to a population of cells that retains stem cell properties. The unique behavior of slow- and fast-cycling HFSC is suggested to depend on differential exposure to quiescent and activating niche cues. Given their isolated localization in the bulge pocket, Bu-SC are exposed only to quiescent-maintaining signals through autocrine, as well as paracrine, signaling from nearby blood vessels and neurons. After the first round of hair generation, differentiated transit-amplifying cells also provide cues to maintain quiescence among Bu-SC [135, 136]. Capillary networks are present in the region above the bulge, and these plexi might influence hair cycling. Interestingly, when angiogenesis is inhibited, anagen induction is delayed, suggesting that angiogenic factors might regulate HFSC activity through vascular-HFSC interactions [137].

Cx43 is the most highly expressed Cx protein in the skin, predominantly in melanocytes, dermal papillae, and keratinocytes, and is involved in the process of HFSC renewal, regeneration, and wound healing [138]. In early mouse development, Cx43 was found in all of the stratified layers of the epidermis. Cx43 is also implicated in maintaining undifferentiated, multipotent skin stem cells. Compared to WT mice, HFSC of newborn Cx43^−/−^ mice exhibit less migration, coupling, and potential, suggesting that Cx43 plays an important role in maintaining undifferentiated and multipotent stem cell niche in the skin [139].

In the hair follicle, adhesion molecules were shown to play an important role in epidermal and hair follicle development and maintenance. E-cadherin ablation in adult epidermis leads to a severe differentiation defect and to decreased proliferation of hair follicle progenitor cells [140]. In addition, in mouse epidermal cells, E-cadherin regulates GJIC via post-translational regulation of Cx43, suggesting that Cx43 and E-cadherin act synergistically for hair follicle maintenance [141, 142].

In acute normal skin injury, Cx43 mRNA level and protein expression are reduced during the entire wound-healing process and this reduction is associated with ECM remodeling, proliferation, and migration of keratinocytes and fibroblast, and regulation of inflammatory responses through cytokines, chemokines, and growth factors [143, 144, 145•]. Although downregulation of Cx43 has been proposed as the main mechanism accelerating the process of acute wound healing [146–148], this dogma has been challenged. Ghatnekar and coworkers showed that Cx43 protein levels are unchanged during acute wound healing [149]. These authors argued that the loss Cx43 CT disrupts the interaction between ZO-1 and Cx43. This leads to a shift of connexon distribution from non-junctional pools to association in GJ, which increases GJ size, whereas the number is reduced [150]. Conversely, in chronic injury, such as ulcers, Cx43 expression is increased near the border of the wound [148, 151].

## Summary and Conclusions

Based on the ubiquity of its expression and the multiple modes of action (GJ and non-GJ), Cx43 is a crucial regulator of stem cell behavior in their respective niches. In this review, we have discussed the known multifaceted roles of Cx43 during development, as well as during adult tissue homeostasis and injury-induced regeneration in different stem cell niches. The synergistic interaction of Cx43 with other junctional and cytoskeleton proteins indicates that beside its junctional role, as either a channel or a hemi-channel, Cx43 also functions as a complex stabilizer at adhesion sites in the regulation of stem cell behavior. It is more and more accepted that most of the unique features, interaction sites, and regulatory domains of Cx43 are attributed to its CT. Thus, it is important to further explore the role of Cx43 CT in the regulation of stem cell behavior and niche maintenance.

We have also highlighted that adult HSC and NSC lie in a vascular niche and that stem cells in other niches may also be regulated by surrounding vasculature. It is important to continue investigating how the close contact of stem cells with the vasculature regulates stem cell behavior. Although we have a substantial amount of data pertaining to the contribution of Cx43 in the maintenance of stem cell niches starting from embryonic development to normal tissue homeostasis and post-injury regeneration in adults, most of these studies remain predominantly descriptive. We still have a long way to go to completely understand the mechanisms by which Cx43 regulates stem cells.

The lethality of Cx43^−/−^ mice at birth makes it complex and challenging to study the role of Cx43 post-natally. However, several conditional KO mouse models have been developed in an attempt to address the role of Cx43 in the regulation of stem cell niches in both physiological and pathophysiological conditions. These models have also provided information enabling the optimization of in vitro systems to study and manipulate the precise role of Cx43, which might ultimately be useful in clinical applications. In the stem cell field, we are all working toward the development of regenerative therapies to repair injury and delay the effects of aging. Understanding the functional roles and the mechanisms of action of Cx43 in bone, neural and skin stem cell niches will provide insights needed for regenerative medicine.
